# 29 Reliability Testing of the Preschool Life Impact Burn Recovery Evaluation Short Forms

**DOI:** 10.1093/jbcr/iraf019.029

**Published:** 2025-04-01

**Authors:** Madeleine McGwin, Pengsheng Ni, Khushbu Patel, Alexandra Gladstone, Vincent Basas, Yasameen Farahvash, Ludwik Branski, Michael Murphy, Frederick Stoddard, Mary Slavin, Tina Palmieri, Jeffrey Schneider, Lewis Kazis, Colleen Ryan

**Affiliations:** Shriners Children’s - Boston; Boston University School of Public Health; Boston Medical Center; Northeastern University; Shriners Children’s - Boston; Shriners Children’s Northern California; University of Texas Medical Branch; Shriners Children’s - Boston; Harvard Medical School; Spaulding Rehabilitation Hospital; University of California, Davis and Shriners Children’s; Spaulding Rehabilitation Hospital, Harvard Medical School; Boston University; Shriners Children’s - Boston and Massachusetts General Hospital

## Abstract

**Introduction:**

The Preschool Life Impact Burn Recovery Evaluation (PS- LIBRE1-5) is a well-established psychometrically sound Computer Adaptive Test (CAT). Short forms (SF) were developed for dissemination in environments where platforms for administering CATs are not available. The aim of the present study is to assess the reliability the PS-LIBRE1-5 SFs within the target population of preschool children, 1 to 5 years, living with burn injuries.

**Methods:**

There are eight PS-LIBRE1-5 domains; Physical Functioning, Communication and Language, Emotional Well-Being, Mood, Anxiety, Peer Relations, Peer Acceptance and Play. Three of the domains: Emotional Well-Being, Communication and Language, and Physical Functioning were suitable for the creation of short forms given their lengthy item pools. Data was obtained from parents/guardians of children 1 to 5 years of age, inclusively, with burn injuries. Parents were administered PS-LIBRE1-5 SFs for a baseline and retest assessment. Retest was administered within 2 to 4 weeks of completion of baseline. Internal consistency reliability of the items at baseline was assessed using Cronbach alpha statistics and Test-retest reliability was assessed using Pearson correlation coefficients.

**Results:**

Test-retest reliability of the PS-LIBRE1-5 SFs was assessed using data from a sample of 263 consented parents of burn survivors, ages 1 to 5 years. 95 parents completed both data points and were included in the test-retest reliability sample. Of these 95 parents, child age was 2.5 ± 1.1 years at survey completion. Mean TBSA burned was 4.5% ± 8.5% and average time since burn was 5.8±9.9 months. Time between baseline and retest surveys was 0.7 months. Cronbach alphas ranged from 0.88 to 0.91 for each domain. Pearson correlation statistics between the test and retest was 0.73 (p< 0.0001) for Emotional well-being, 0.83 (p< 0.0001) for Physical Functioning, and 0.91(p< 0.0001) for Communication and Language.

**Conclusions:**

The PS-LIBRE1-5 SFs demonstrated acceptable reliability in the present analysis. Reliability of this assessment is important in determining the relative precision of the assessment. Future work can assess the responsiveness to change of the SF’s.

**Applicability of Research to Practice:**

PS-LIBRE1-5 SFs of Emotional Well-Being, Communication and Language, and Physical Functioning are reliable for use in the assessment of burn recovery in preschool-aged children. These instruments can be made available in clinical settings or future studies where access to CAT technology is not available.

**Funding for the Study:**

This work was funded by Foundation Funding (Grant #79136) and partial support from the National Institute on Disability, Independent Living, and Rehabilitation Research (Grant #90DPBU0008).

